# Allergy by Proxy to Penicillins Presenting as Recurrent Idiopathic Anaphylaxis Within the Household: A Case Report

**DOI:** 10.1111/cod.70014

**Published:** 2025-09-22

**Authors:** Adrien Bénic, Evangéline Clark, Pascal Demoly, Anca Mirela Chiriac

**Affiliations:** ^1^ Division of Allergy, Department of Pulmonology Hospital Arnaud de Villeneuve, University Hospital of Montpellier Montpellier France; ^2^ Department of Allergy Hospital Layné Mont‐de‐Marsan France; ^3^ IDESP, UMR UA11, Univ. Montpellier—INSERM Montpellier France

**Keywords:** anaphylaxis, beta‐lactams, case report, drug hypersensitivity, hypersensitivity, immediate, penicillins

## 1 Case Report

A 38‐year‐old man with a history of allergic rhinitis experienced seven episodes of anaphylaxis at home, one of which progressed to anaphylactic shock. He denied any medication use, ingestion of specific foods or Hymenoptera stings prior to these episodes.

The initial evaluation was conducted in an allergist's office. The baseline serum tryptase level was 4.10 μg/L. Skin testing for common food allergens yielded negative results, with an appropriate positive histamine control.

The patient was subsequently referred to our unit for further assessment. On detailed re‐questioning, he reported that his anaphylactic reactions consistently coincided with periods during which his children were receiving penicillin therapy, and he was able to identify specific triggers for two of these episodes (Table 1).

**TABLE 1:** Patient's immediate‐type hypersensitivity reactions resulted from indirect exposure to penicillins.

Allergen	Trigger	Onset time	Clinical presentation	Acute serum tryptase	Treatment	Resolution time
Amoxicillin	Licking his child's spoon containing traces of amoxicillin syrup	5 min	Palmo‐plantar itching Head itching Facial angioedema Dyspnea	Not measured	Oral corticosteroids	A few hours
Amoxicillin	Drinking from his child's glass containing traces of a crushed amoxicillin tablet	10 min	Generalised angioedema Vomiting Dyspnea Loss of consciousness	Not measured	None	A few hours

Serum‐specific IgE testing for beta‐lactams (ImmunoCAP, Thermo Fisher Scientific/Phadia, Uppsala, Sweden) was positive only for amoxicillin (0.26 kU_A_/L). Total serum IgE was not assessed.

Skin testing for beta‐lactams was performed in accordance with established guidelines [1]. Skin prick tests produced immediate positive reactions to amoxicillin (0.2 mg/mL; 1:100) and ampicillin (2 mg/mL; 1:10), whereas intradermal tests yielded an immediate positive reaction to penicillin G (10,000 IU/mL; undiluted), confirming sensitisation to penicillins.

The absence of hypersensitivity to alternative beta‐lactams—specifically the cephalosporins cefuroxime and ceftriaxone—was demonstrated by negative skin tests (20 mg/mL; undiluted) and negative drug provocation tests at daily therapeutic doses of 1000 and 2000 mg, respectively.

## 2 Discussion

‘Allergy by proxy’ refers to hypersensitivity reactions arising from unintentional exposure to an allergen used by another individual. Such exposure may occur directly, through physical contact with the person carrying the allergen, or indirectly, via contact with allergen‐contaminated objects [2]. A well‐documented example is kissing‐induced allergy syndrome, in which oral exposure to amoxicillin‐containing saliva triggers an allergic reaction [3]. Similarly, anaphylaxis has been reported during sexual intercourse as a result of vaginal exposure to penicillin‐containing semen [4].

In this case, the patient experienced severe IgE‐mediated hypersensitivity reactions attributable to ‘allergy by proxy’, resulting from indirect exposure to amoxicillin via contact with items contaminated during his children's penicillin therapy. In the absence of any prior personal use of beta‐lactams, sensitization to penicillins likely occurred through repeated cutaneous or mucosal exposure to penicillin residues within the household.

The patient was advised to strictly avoid all penicillins and to remain vigilant regarding their use by household members. Considering the patient's history of anaphylactic reactions to trace amounts of penicillins, along with the regular use of these antibiotics by his children at home, an emergency kit containing an epinephrine auto‐injector was prescribed for potential use in the event of accidental re‐exposure. If beta‐lactam therapy is required in the future, only cephalosporins with documented tolerance—specifically cefuroxime and ceftriaxone—should be considered. This observed tolerance is consistent with current evidence indicating that cross‐reactivity between penicillins and cephalosporins with different side chains is rare [1].

In conclusion, physicians should systematically evaluate both environmental and interpersonal exposures when investigating recurrent, unexplained allergic reactions. The identification of hidden allergen sources is crucial to prevent potentially life‐threatening recurrences. Given their widespread use and propensity to induce anaphylaxis, penicillins should be consistently considered as potential culprits in such scenarios.

## Conflicts of Interest

The authors declare no conflicts of interest.

## Data Availability

The data that support the findings of this study are available from the corresponding author upon reasonable request.
